# The Night Side of Blood Pressure: Nocturnal Blood Pressure Dipping and Emotional (dys)Regulation

**DOI:** 10.3390/ijerph17238892

**Published:** 2020-11-30

**Authors:** Maria Casagrande, Francesca Favieri, Viviana Langher, Angela Guarino, Enrico Di Pace, Giuseppe Germanò, Giuseppe Forte

**Affiliations:** 1 Dipartimento di Psicologia Dinamica e Clinica, Università di Roma “Sapienza”, 00185 Roma, Italy; viviana.langher@uniroma1.it; 2 Dipartimento di Psicologia, Università di Roma “Sapienza”, 00185 Roma, Italy; francesca.favieri@uniroma1.it (F.F.); angela.guarino@uniroma1.it (A.G.); enrico.dipace@uniroma1.it (E.D.P.); g.forte@uniroma1.it (G.F.); 3 Dipartimento di Scienze Cardiovascolari, Respiratorie, Nefrologiche e Geriatriche, Università di Roma “Sapienza”, 00815 Roma, Italy; ncgerman@tin.it

**Keywords:** blood pressure, dipping status, anger, ambulatory blood pressure monitor

## Abstract

*Introduction:* The dipping phenomenon is a physiological drop in blood pressure (around 10–20%) during sleep and represents an event related to the circadian blood pressure trend. This phenomenon, in some cases, is characterized by some alterations that can be expressed by an increase (extreme dipping), a decrease (non-dipping), or a reverse (i.e., higher blood pressure during sleep compared to awake state; reverse-dipping) physiological decline of blood pressure. Few studies focused on the association between the circadian variation of blood pressure and psychological variables, although this information could help understanding how psychological characteristics (e.g., emotional regulation or dysregulation) interact with individuals’ physiological processes. Given the association between emotional dysregulation and essential hypertension, this study aimed to investigate the relationship between alexithymia and dipping status in a sample of healthy and hypertensive adults in the absence of other medical conditions. *Methods:* Two hundred and ten adults took part in the study and were classified, according to ambulatorial blood pressure measure (ABPM), into three groups: dippers (*n* = 70), non-dippers (*n* = 70), and extreme dippers (*n* = 70). The participants completed a socio-demographic and anamnestic interview and the Toronto Alexithymia Scale-20 (TAS-20). *Results:* The ANOVAs on the TAS-20 subscales showed that the groups differed in the difficulty identifying feelings and difficulty describing feelings. In both the subscales, dippers showed lower scores than non-dippers and extreme dippers. The ANOVA on the global score of TAS-20 confirmed that dippers were less alexithymic than both extreme dippers and non-dippers. *Conclusions:* This study confirms that some psychological factors, like alexithymia, could represent a characteristic of patients who fail to exhibit an adaptive dipping phenomenon. Moreover, an association between an excessive reduction of BP (extreme dipping) or a lack of the decrease of BP during sleep (non-dipping) and a worse emotional regulation, considering alexithymia construct, was highlighted for the first time, confirming the relevant role of the emotional process in the modulation of an essential psychophysiological process such as the circadian variation of BP.

## 1. Introduction

The circadian variation of sympathetic activity during night-time is physiologically accompanied by an increased vagal tone and a marked reduction in heart rate, cardiac output, and total peripheral resistance. As a consequence of these autonomic variations, blood pressure (BP) drops during the bed-rest period. Typically, the physiological night-time drop or “dip” of BP ranges between 10 and 20% compared with daytime BP [1,2,3]. However, in some cases, this phenomenon may be characterized by an atypical variation.

According to recent hypertension guidelines, assessment of nocturnal BP values and dipping status is enabled by 24-h ambulatory blood pressure monitoring (ABPM), which allows the classification of patients in four different circadian BP phenotypes: (a) dipping: physiological decrease of 10–20%; (b) non-dipping: reduction of night-time BP less than 10%; (c) reverse dipping: a rise of night-time BP; and (d) extreme dipping: a night-time BP decrease higher than 20% [4].

The physiological dipping pattern appears to be a protective factor for cardiovascular health [5]. Conversely, the circadian BP phenotypes of extreme dipping and non-dipping appear to be associated with different risks of organ damage, cardiovascular outcomes, and higher mortality [3,6,7,8].

It is well known that many psychological factors play an important role in the development and maintenance of altered BP and essential hypertension. The studies focused on hypertension have found an association of hostility, depression, anxiety, coping strategies, and emotional dysregulation with alteration of the normal level of blood pressure [9,10,11,12,13,14]. However, the causes responsible for nocturnal blood pressure variations remain unclear; some studies have highlighted the possible involvement of some psychological variables, such as anger and hostility [15,16,17], depression [18], low perceived social support [19], social discrimination [20], and work-related stress [21]. However, to our knowledge, no study analyzed the possible role of emotional dysregulation in dipping alteration.

For the first time, the current study investigates the association between alexithymia, an emotional dysregulation construct, and the BP dipping status. Previous evidence reported higher emotional dysregulation in people with essential hypertension [12,13]. Moreover, both non-dipping and extreme dipping conditions were considered as possible cardiovascular risk factors [1,8,17,22,23]. Accordingly, it was expected that both extreme and non-dipping phenotypes would show higher alexithymia scores than dippers.

## 2. Method

### 2.1. Participants

Two hundred and ten participants were recruited at the First Medical Clinic of the Policlinico Umberto I of the University of Rome “Sapienza”. A cardiologist provided information about the hypertensive status and dipping conditions. The participants were divided into three groups, according to their bed-time drops of BP: dippers (*n* = 70) who had a dipping ratio between 0.80 and 0.90; non-dippers (*n* = 70) who presented a dipping ratio higher than 0.90; and extreme dippers (*n* = 70) who showed a dipping ratio lower than 0.80.

The inclusion criteria for the study were: (a) age between 40 and 75 years and (b) dipping classification performed by a cardiologist after a 24-h ABPM. The exclusion criteria were a diagnosis of chronic or severe medical and psychiatric pathologies (such as cancer, diabetes, cardiac or neurological pathologies, and psychiatric disorders).

The main characteristics of the samples are shown in Table 1.

### 2.2. Assessment Tools

#### 2.2.1. Physiological Measures

Blood pressure office evaluation: systolic (SBP) and diastolic Blood Pressure (DBP) and heart rate were recorded by using an automatic electronic sphygmomanometer (Omron; Kyoto, Japan) validated for self-measurement (“Personal Check” PIC). Blood pressure measurement was performed according to the European guidelines for hypertension [24].

Weight status assessment: weight and height, assessed by a balance and a stadiometer, were used to calculate the body mass index (BMI; kg/m^2^), an indirect estimate of the individual’s body fatness.

#### 2.2.2. Ambulatory Blood Pressure Monitors (ABPM)

The 24 h BP was measured with the Takeda ABPM monitor (TM-2430; Takeda Pharmaceutical Company Limited; Tokyo, Japan). The ABPM was set to obtain BP recordings at 15 min intervals during the wake (time range of daily blood pressure: 07:00 to 22:00) and 30 min intervals during sleep (time range of night blood pressure: 22:00 to 07:00) [25]. According to the practice guidelines for ambulatory BP measurement [25], different criteria were adopted to have BP measurement as reliable as possible. The ABPM sessions were performed during a weekday before the psychological evaluation, and participants were instructed to attend their usual daily activities but to hold their arm stationary during BP readings. Additionally, they filled in a log on the activities carried out during the BP registration day, and they reported the time of both sleep onset and awakening.

Furthermore, they recorded whether they were out of bed during the night (e.g., to use the bathroom) and the relative awake time. This strategy allows excluding the “out-of-bed” records of the ABPM from the data. Another exclusion criterion for the measurements was the presence of SBP data greater than 240 mmHg or lower than 70 mmHg or DBP data higher than 150 mmHg or lower than 40 mmHg. The ABPM dataset included in the analysis should have at least two-thirds of the SBP and DBP measurements during both the daytime and the night-time periods [25]. However, no participants in the study reported invalid data. The ABPM recordings allow calculating the mean arterial pressure (MAP) of wake and sleep through the formula MAP = (SBP + (2 × DBP))/3, and dipping ratio was assessed by the ratio between sleep MAP and wake MAP [26].

### 2.3. Socio-Demographic and Anamnestic Information

Demographic data (age, gender, years of education), lifestyles (smoking and alcohol consumption), medical, and psychiatric information were collected for each patient by face-to-face interviews.

### 2.4. Questionnaires

#### 20-Item Toronto Alexithymia Scale (TAS-20)

TAS-20 [27,28]: a 20-item self-report questionnaire to evaluate alexithymia. The test assessed three different dimensions of alexithymia: difficulty identifying feelings (DIF), difficulty describing feelings (DDF), and externally oriented thinking (EOT). A 5-point Likert scale classifies the responses (1 = strongly disagree, 5 = strongly agree), and the scores range from 20 to 100. Higher scores in the three dimensions of TAS-20 and TAS-20 global score identify high alexithymic levels.

### 2.5. Procedure

The study was conducted according to the Declaration of Helsinki and was approved by the Local Ethics Committee (Department of Dynamic and Clinical Psychology—“Sapienza” University of Rome; number: 0001166). After the informed consent was signed, each participant was subjected to office blood pressure assessment; then, weight and height were recorded. Finally, the participants completed the socio-demographic and anamnestic interview and the TAS-20 questionnaire. After this procedure, a cardiologist gave the eventual diagnosis of essential hypertension and classified the dipping status according to the ABPM registration.

### 2.6. Data Analyses

One-way analyses of variance (ANOVAs) were carried out considering the group as the independent variable (non-dippers, dippers, extreme dippers) and the different socio-demographic (age, years of education), physiological (SBP, DBP, MAP day, MAP night, night/day MAP ratio, BMI), and lifestyle (smoking and alcohol consumption) dimensions as dependent variables.

Finally, ANOVAs on the TAS-20 subscales and TAS-20 global scores were conducted. Planned comparisons were used to analyze significant effects.

The Chi-squared test (χ^2^) was adopted to estimate the differences in the percentage of normotensive, untreated hypertensive, and treated hypertensive participants in the three groups. For all the statistical analyses, the level of significance was accepted at *p* ≤ 0.05. Statistical analyses were performed through the Statistica Software v.10.0 (StatSoft.inc., Tulsa, OK, USA).

## 3. Results

Table 1 shows the differences between groups in the main demographic and physiological characteristics.

**Table 1:** Means (±SD) of the main characteristics of the three groups of participants.

	Non-Dippers	Dippers	Extreme Dippers	F/χ^2^	*p*
*n* (Female/Male)	70 (35/35)	70 (36/34)	70 (36/34)		
Age	59.99 (8.46)	56.10 (10.13)	53.69 (9.04)	8.29	0.001
Years of education	11.72 (3.23)	12.66 (3.95)	13.56 (4.22)	3.05	0.06
Body mass index (BMI)	26.64 (4.99)	25.88 (4.28)	26.47 (3.69)	<1	0.56
Systolic blood pressure (SBP)	140.61 (22.50)	141.82 (18.68)	141.67 (18.62)	<1	0.93
Diastolic blood pressure (DBP)	88.26 (12.30)	91.57 (12.08)	92.18 (10.50)	2.26	0.11
Mean arterial pressure day (MAP)	97.66 (10.08)	98.30 (9.00)	101.82 (8.16)	4.24	0.02
Mean arterial pressure night (MAP)	92.15 (9.89)	83.00 (7.40)	76.67 (6.53)	65.13	0.0001
Dipping ratio MAP	0.95 (0.03)	0.85 (0.03)	0.76 (0.03)	675.4	0.0001
Smoking cigarettes (number per day)	0.30 (0.75)	0.51 (0.88)	0.32 (0.67)	1.50	0.23
Alcohol consumption (number of glasses per day)	0.36 (0.48)	0.40 (0.52)	0.31 (0.53)	<1	0.60
Hypertensive conditions (*n* (%))				16.98	0.02
Normotensive	14 (39)	9 (25)	13 (36)		
Hypertensive untreated	11 (16.7) ^a^	23 (34.8)	32 (48.5) ^a^		
Hypertensive treated	45 (41.7) ^b^	38 (35.2)	25 (23.1) ^b^		
Alexithymic condition				19.34	0.001
Non-alexithymic	46 (32.1)	55 (38.5)	42 (29.4)		
Moderately alexithymic	15 (32.6)	15 (32.6)	16 (34.8)		
Alexithymic	9 (42.9) ^c^	0 (0.0) ^cd^	12 (57.1) ^d^		

χ^2^ significant differences between groups = ^a^: (χ^2^ = 7.95; *p*< 0.005); ^b^: (χ^2^ = 3.84; *p*< 0.05); ^c^: (χ^2^ = 8.49; *p* < 0.003); ^d^: (χ^2^ = 11.12; *p* < 0.001).

### 3.1. Demographical, Physiological, and Lifestyle Variables

The ANOVA on age showed significant differences between groups (F_2,207_ = 8.29; *p* = 0.001; *p*_ƞ_^2^ = 0.07); non-dippers were older than both dippers (F_1,207_ = 6.19; *p* = 0.002) and extreme dippers (F_1,207_ = 16.27; *p* = 0.0001). No differences were highlighted between dippers and extreme dippers (F_1,207_ = 2.39; *p* = 0.13). Considering years of education (F_2,207_ = 3.05; *p* = 0.06), BMI (F_2,207_ = 0.59; *p* = 0.56), systolic blood pressure (F_2,207_ = 0.08; *p* = 0.93), diastolic blood pressure (F_2,207_ = 2.26; *p* = 0.11), cigarette consumption (F_2,207_ = 1.50; *p* = 0.23), and alcohol consumption (F_2,207_ = 0.52; *p* = 0.60), no significant differences were observed among the groups (see Table 1).

The ANOVAs on the two indices of MAP (day and night) showed significant differences.

**Figure 1:**
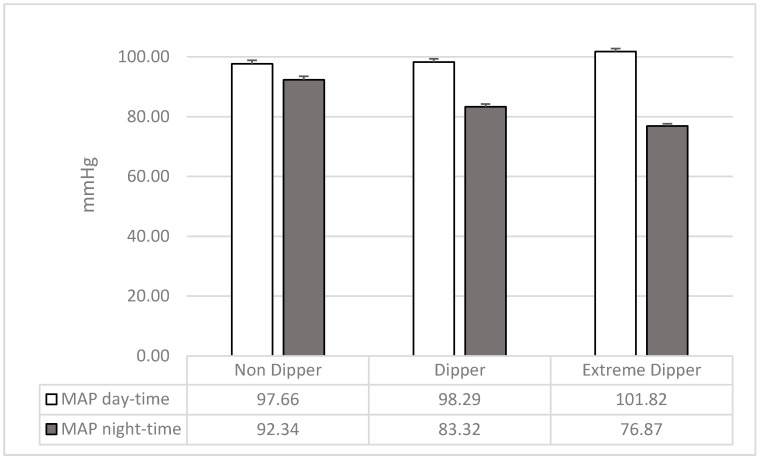
Mean and SD of mean arterial pressure (MAP) of wake and sleep in the three groups of participants.

Considering MAP of day time (F_2,207_ = 4.24; *p* = 0.02; *p*_ƞ_^2^ = 0.04) extreme dippers showed higher values than dippers (F_1,207_ = 5.51; *p* = 0.02) and non-dippers (F_1,207_ = 7.23; *p* = 0.01), while dippers and non-dippers were not different (F_1,207_ = 0.12; *p* = 0.73). Considering MAP of night time (F_2,207_ = 65.13; *p* = 0.0001; *p*_ƞ_^2^ = 0.39), extreme dippers showed lower values than both dippers (F_1,207_ = 21.53; *p* = 0.0001) and non-dippers (F_1,207_ = 128.83; *p* = 0.0001); and dippers showed lower values than non-dippers (F_1,207_ = 45.03; *p*= 0.0001) (Figure 1; see also Table 1).

### 3.2. Alexithymia

Table 2 shows the TAS-20 scores of the three groups.

**Figure 2:**
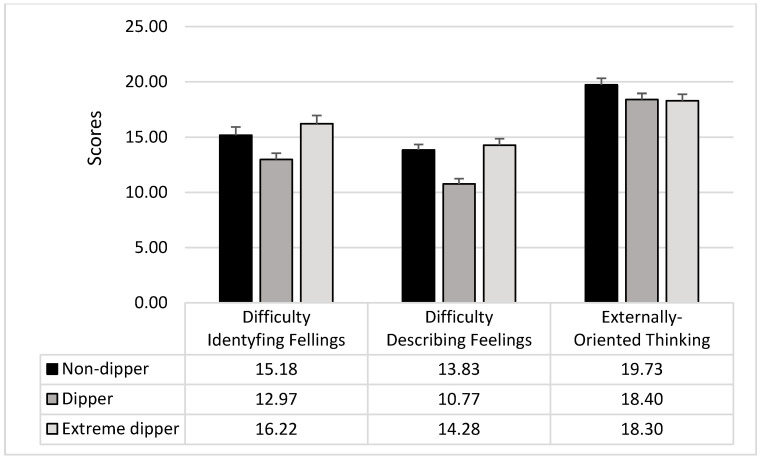
Mean and SD of the TAS-20 subscales in the three groups of participants.

ANOVAs on the TAS-20 subscales showed significant differences between the groups in the difficulty identifying feelings (F_2,207_ = 5.63; *p* = 0.005; *p*_ƞ_^2^ = 0.05) and the difficulty describing feelings (F_2,207_ = 13.56; *p* = 0.0001; *p*_ƞ_^2^ = 0.12). Dippers showed lower scores in both the TAS-20 subscales than non-dippers (DIF: F_1,207_ = 5.02; *p* = 0.03; DDF: F_1,207_ = 17.41; *p* = 0.0001; see Figure 1) and extreme dippers (DIF: F_1,207_ = 10.79; *p* = 0.002; DDF: F_1,207_ = 22.94; *p* = 0.0001; see Figure 1); non-dippers and extreme dippers were not different (DIF: F_1,207_ = 1.09; *p* = 0.30; DDF: F_1,207_ = 0.38; *p* = 0.54; see Figure 2).

Externally oriented thinking did not result different among the three groups (F_2,207_ = 1.91; *p* = 0.15).

**Figure 3:**
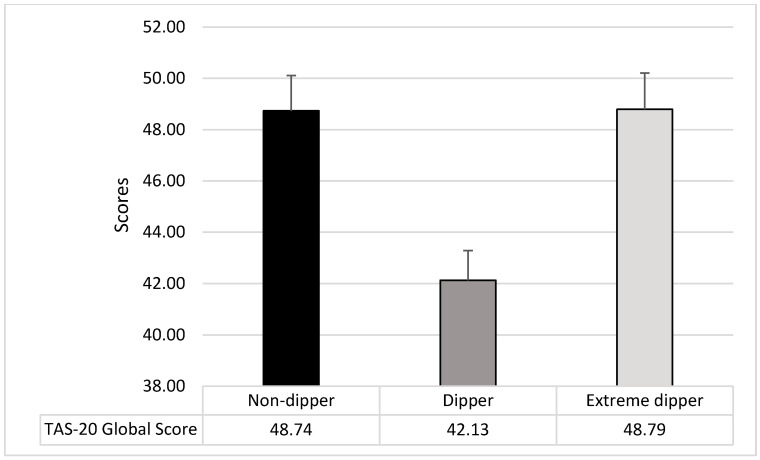
Mean and SD of the TAS-20 Global Score in the three groups of participants.

The ANOVA on TAS-20 global score showed a significant difference among groups (F_2,207_ = 8.45; *p* = 0.0003; *p*_ƞ_^2^ = 0.08); dippers showed lower levels of alexithymia than both non-dippers (F_1,207_ = 12.57; *p* = 0.0005) and extreme dippers (F_1,207_ = 12.77; *p* = 0.0005), while there were no differences between non-dippers and extreme dippers (F_2,207_ = 0.001; *p* = 0.98) (see Figure 3).

Since age was significantly different between groups, it was introduced as covariates in the analyses; however, no substantial differences were found. ANOVAs confirmed all significant differences.

### 3.3. Alexithymia in Normotensive and Hypertensive Subgroups

In the hypertensive patients, the ANOVAs showed significant differences in the difficulty identifying feelings (F_2,171_ = 6.28; *p*= 0.002; *p*_ƞ_^2^ = 0.06), difficulty describing feelings (F_2,171_ = 12.90; *p* = 0.0001; *p*_ƞ_^2^ = 0.12), externally oriented thinking (F_2,171_ = 3.48; *p*= 0.03; *p*_ƞ_^2^ = 0.04) and global score (F_2,171_ = 8.90; *p* = 0.001; *p*_ƞ_^2^ = 0.10). Planned comparison confirmed that extreme dippers and non-dippers reported higher TAS-20 scores compared to dippers in difficulty identifying feelings (dipper vs non-dipper: F_1,171_ = 6.88; *p* = 0.01; dipper vs extreme dipper: F_1,171_ = 11.23; *p* < 0.0001), difficulty describing feeling (dipper vs non-dipper: F_1,171_= 17.26; *p* = 0.0001; dipper vs extreme dipper: F_1,171_ = 21.04; *p* = 0.0001) and the global score (dipper vs non-dipper: F_1,171_= 15.27; *p* = 0.001; dipper vs extreme dipper: F_1,171_ = 11.08; *p* = 0.001),

No differences were reported in normotensive participants (*p* > 0.05).

## 4. Discussion

The dipping phenomenon is a physiological characteristic of the circadian blood pressure rhythm [1]. Alterations in this pattern have important consequences for cardiovascular health. Accordingly, dipping status represents a more reliable predictor of cardiovascular morbidity and mortality than the diurnal BP [29]. Many psychological dimensions are associated with alteration in BP dipping [17,30]. For the first time, the present study highlights a relationship of the dipping pattern with another psychological trait, i.e., alexithymia. High levels of alexithymia, expressed by difficulty identifying and describing feelings, were associated with extreme and non-dipping patterns in a sample, including hypertensive and normotensive people.

Previous research evidenced that difficulty identifying and describing feelings characterize people with essential hypertension and adverse cardiovascular outcomes [12,13,14]. Our results provide additional information to this complex relationship, confirming that individuals with absent or excessive blood pressure dipping present higher emotional dysregulation than individuals with physiological dipping. This result suggests that difficulty in identifying and describing feelings, which represent a typical pattern of problematic processing and managing of emotions and emotional stimuli, could be considered a possible risk factor or a marker of the alteration of circadian BP rhythm.

In interpreting these outcomes, it must be considered that the autonomic nervous system (ANS) plays a crucial role in this relationship because it appears to be the main mediator of the circadian variation in blood pressure, and it is related to emotional response [31]. In particular, the sympathetic nervous system activity generates the difference between sleep-time and wake-time blood pressure. Moreover, primary autonomic failure and very low sympathetic and parasympathetic activities are associated with a high non-dipping BP incidence. This situation suggests that an altered dipping process is due to the inability to modulate autonomic tone rather than to the failure to inhibit sympathetic activity during sleep [32]. According to this assumption, the increase of cardiovascular risk associated with non-dipping status seems to be due to the difficulty in physiological recovering from the typical demand-driven elevations of blood pressure during the waking hours of the day [33]. Therefore, the non-dipping status appears characterized by a disruption of the circadian autonomic balance [34]. Moreover, an increase in sympathetic nervous system activity results in cognitive and psychological alterations [35,36,37,38], also involving emotional regulation ability.

Alexithymia is characterized by somatosensory amplification and hyperarousal, and these features disturb the affective arousal induced by external stimuli [39]. The alteration of the physiological response associated with emotional stimuli, and the fact that some of the areas involved in the elaboration of these stimuli (e.g., amygdala, limbic system) are also involved in the autonomic regulation of circulation responses to stress events [40], would confirm the physiological connection between alexithymic characteristics and the circadian rhythm alteration of blood pressure.

The results concerning externally oriented thinking are in line with this assumption. In our sample, no differences between groups were found in this alexithymic dimension. This result could occur because externally oriented thinking refers more to a cognitive level of emotional process, whereas difficult to identify and describe feelings are closely related and refer to the subjective experience of emotions that are strictly associated with the hyperactivation of the sympathetic nervous system and the consequent hyperarousal [41,42].

When the relationship between the dipping status and alexithymia was separately examined in hypertensive and normotensive participants, a higher emotional dysregulation in extreme dipper and non-dipper individuals was confirmed only in hypertensive patients. This finding suggests that alexithymia may be associated with an alteration of the cardiovascular system in hypertensive participants, which would further compromise hypertension. Moreover, this result could indicate that the well-known relationship between hypertension and alexithymia, e.g., [12,13,14], is restricted only to hypertensive patients that show an anomalous dipping pattern (i.e., non-dippers and extreme dippers). However, further studies with larger samples are needed to confirm this hypothesis. In fact, the data do not allow any conclusions to be drawn about normotensive people. The small number of normotensive individuals does not allow for an adequate subdivision of participants based on the dipping trend.

The findings of this study are interesting because they were highlighted also controlling a possible influence of demographic (such as gender and years of education), physiological (BMI), and lifestyle (cigarette and alcohol consumption) factors that did not differ among the groups. Only the age was different in the three groups, but the observed results were maintained by introducing this variable as a covariate in the analyses. Moreover, dippers were not different from non-dippers and extreme dippers in the prevalence of normotensive, hypertensive untreated, and hypertensive treated participants. A previous study [12] reported a relationship between alexithymia and uncontrolled hypertension, supporting the hypothesis that higher emotional dysregulation is associated with a high cardiovascular risk. This study reported a non-linear pattern between alexithymia and circadian variation of BP, confirming the role of alexithymia as a possible independent risk factor for cardiovascular disease and a possible psychological marker of BP alterations.

Generally, the importance of controlling the role of the psychological aspects in the circadian variation of BP and the dipping phenomenon was reinforced by the present findings. Moreover, contrary to previous studies that considered dipping status as a dichotomic or continuous phenomenon, our results underlined the importance of a multi-dimensional classification, which considers the different forms of nocturnal blood pressure variation [18].

Although the results are promising, the current study presents some limitations, such as the small sample size. This factor underlines the need to replicate these findings in larger community samples to generalize these results to the general population. Another limit was represented by the use of the ABPM for the assessment of BP. ABPM is characterized by a poor reproducibility of measurements, as highlighted by some authors [43]. However, all precautions were taken to avoid errors, such as using a short bed-time range (00:00–06:00) and using mean arterial pressure (MAP) for the dipping classification [25].

The absence of drug treatment analysis represents another limit because we do not know how antihypertensive treatment could have affected the BP circadian variations. It would be useful to analyze this aspect, which can mediate the relationship between psychological and physiological aspects [12,17]. The last limit to consider is the absence of a group of participants with reverse dipping patterns (i.e., characterized by increased BP during sleep; [18]). This pattern would be associated with high cardiovascular risk and increased mortality [1], and to analyze if and how the psychological variables influenced this pattern appears important.

## 5. Conclusions

Although the knowledge on the association between blood pressure and psychological factors has increased in the last years, confirming the role of different psychological aspect in different types of hypertension [11,12,17], less is known about the influence of psychological characteristics on the circadian rhythm of BP and the dipping phenomenon.

It would be useful to fully understand how blood pressure interacts with other individual traits, such as psychological dimensions, to improve the diagnosis and treatment of hypertension and related disorders.

Our findings suggest that psychological factors may contribute to the description and classification of patients who fail to exhibit adaptive nocturnal blood pressure dipping. It is interesting to note that the relationship between emotional dysregulation, i.e., difficulty identifying and describing feelings, and excessive BP reduction during sleep (extreme dipping) underlines that some psychological aspects could affect the absence of dipping and a maladaptive increase of the dipping phenomenon. These findings suggest that alterations in the circadian pattern of BP can be linked to emotional dysregulation in a non-linear way that should be further examined.

## Figures and Tables

**Table 2:** Means (±SD) of the TAS-20 scores in the three groups of participants and ANOVAs results.

	Non Dippers	Dippers	Extreme Dippers	F	*p*
DIF	15.18 (6.28)	12.97 (4.89)	16.22 (6.27)	5.63	0.005
DDF	13.83 (4.20)	10.77 (3.93)	14.28 (4.84)	13.56	0.0001
EOT	19.73 (4.95)	18.40 (4.71)	18.30 (4.87)	1.91	0.15
Total score	48.74 (11.43)	42.13 (9.67)	48.79 (11.86)	8.45	0.0003

DIF: difficulty identifying feelings; DDF: difficulty describing feelings; EOT: externally oriented thinking.
